# Mutations in the cell-binding motif of *lam-3/laminin α* reveal hypercontraction behavior and defective sensitivity to levamisole in *Caenorhabditis elegans*

**DOI:** 10.17912/micropub.biology.000485

**Published:** 2021-10-11

**Authors:** Lianzijun Wang, Zhongqiang Qiu, Myeongwoo Lee

**Affiliations:** 1 Department of Biology, Baylor University, Waco, TX 76798, U.S.A

## Abstract

The amino acid sequence Arg-Gly-Asp (RGD) is a cell-binding motif for extracellular matrix proteins. Initially found in fibronectin, the RGD motif is also found in LAM-3/laminin α chain in *C. elegans*. Laminin, a heterotrimeric glycoprotein, is a significant component of the basement membrane. Mutations in laminin subunits disrupt the extracellular matrix hence inhibit cell adhesion. This study aims to characterize the function of the RGD motif in *lam-3*/laminin α. Two mutations, *lam-3* RGE and *lam-3* ΔRGD, were generated. Our analysis of the mutants revealed that the RGD motif is involved in the motility of animals, suggesting that the cell-laminin interaction plays a role in regulating body contraction.

**Figure 1:**
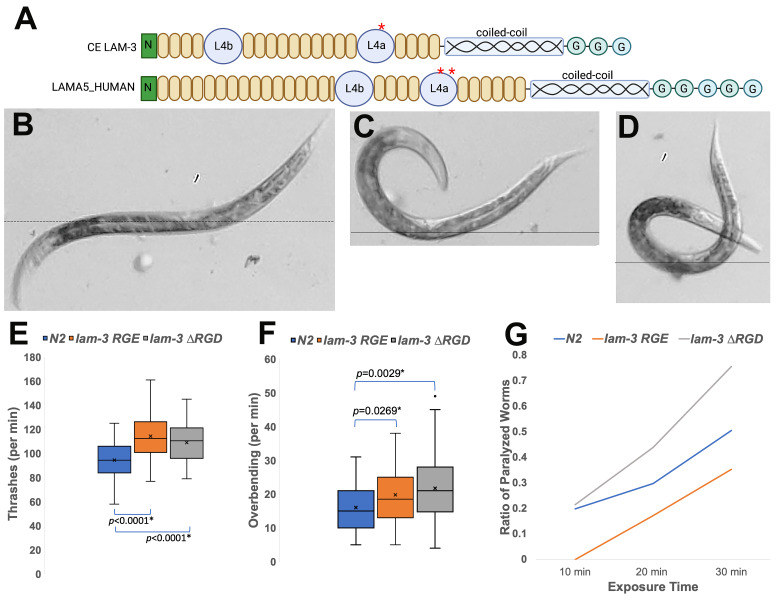
A. Diagram of *C. elegans* LAM-3/laminin α and LAMA5_human (human laminin α5). N in a green rectangle: N-terminal domain; Yellow rounded rectangles: rod-domains composed of EGF repeats; L4a and L4b: laminin IV domain type A and B; helix mark: coiled-coil domain; G: globular domain at C-terminal. The red asterisks indicate the locations of the RGD motif. They were adopted and modified from diagrams (Colognato and Yurchenco 2000; Huang *et al.* 2003). B. N2 thrashing. The position represents the thrashing behavior of *C. elegans* in M9 buffer, which is the coordinated contraction of body wall muscles. A horizontal line across the midpoint helps to visualize the axis to which the anterior and posterior body bends. C. The position shown here demonstrates uncoordinated body bends. This thrashing was counted as a “hook”-shaped overbending, which resulted from either the anterior or posterior end of the nematode contracts or bends for the maximum degree towards the mid-body region. This worm failed to show sinuous or rhythmic bending by having a tendency to pause at a contracted position, shown here. A horizontal line across the midpoint helps to visualize the axis. D. This worm represents a full-length body overbending. This bending results from the hyper-contraction of both the anterior and posterior end simultaneously. It results in overcrossing of the head and tail, as shown in the panel. A horizontal line across the midpoint helps to visualize the axis. E. One-minute thrashing counts. Cross marks in the boxes represent the mean of thrashes. Two mutants, *lam-3* RGE *(kq1464)* and *lam-3* ΔRGD *(kq1461),* have a higher mean of thrashes than that of N2. F. Overbending shown in panels C and D were counted for each strain. One outlier is shown as a dot outside the fourth quartile of *lam-3* ΔRGD *(kq1461)* panel. N = 50. G. Levamisole assay. The percentage of paralyzed animals at three different time points over the course of 30 minutes.

## Description

*Laminin* is a heterotrimeric glycoprotein composed of α, β, and γ subunits, one of the significant components in the basement membrane extracellular matrix (ECM). It plays an essential role in tissue organization such as ECM assembly, cell migration, and cell adhesion (Colognato and Yurchenco 2000). In the ECM, laminin interacts with cell surface receptors such as integrins and dystroglycan, linking ECM to actin cytoskeletons in muscle cells (Gawlik *et al.* 2006; Meinen *et al.* 2007). Among the laminin subunits, several laminin α chains bind to integrins. There are five α chains in humans. LAMA5 (laminin α5) contains the RGD motifs in its laminin IV type A domain (L4a). In *C. elegans*, LAM-3/laminin α possesses an RGD motif in the L4a domain, similar to the RGD location in LAMA5 (Figure 1A). *Dystroglycan* is a non-integrin binding receptor that bridges laminin to the cytoskeleton in skeletal muscle cells. Dystrophin and other transmembrane proteins are collectively identified as the dystrophin-glycoprotein complex that binds to laminins. Deficiencies in dystroglycan complex and laminin are linked to congenital muscular dystrophy (Sciandra *et al.* 2007; Sciandra *et al.* 2015). We surveyed ECM proteins and found an RGD (Arg-Gly-Asp) motif in LAM-3/laminin α (amino acid numbers 1462, 1463, and 1464). The RGD motif was edited to RGE or deleted to study the function of the motif.

In order to characterize the function of the RGD motif of LAM-3, the RGD motif was removed from the *lam-3* locus by using the CRISPR-Cas9 system, which produced a mutant allele *lam-3 (kq1461)*, designated as *lam-3* ΔRGD. The RGD sequence is also changed to RGE (Arg-Gly-Glu), which produces another allele, *lam-3 (kq1464)*, designated as *lam-3* RGE (see Methods section). Previous studies on the ability of laminins to bind to integrins and dystroglycan (Yurchenco *et al.* 2018) hypothesized that mutations of the *lam-*3 RGD motif would result in locomotion anomalies. The thrashing (swimming) behavior in liquid medium has been a well-established and efficient method for measuring motility (Koopman *et al.* 2019). Thrashing is a coordinated contraction; the worm synchronously bends the anterior and posterior parts of body wall muscle cells. Typical thrashing behavior is characterized as worms swinging from one side to the other of the body axis (Figure 1B). Overbending movement occurs when muscle contraction is not harmonious across the body, generating a stop in either a “hook” position (Figure 1C) or an overbent U-shape (Figure 1D) (Ackley *et al.* 2003). When performing the thrashing assays, the number of overbending (Figures 1C and 1D) is not included in the thrashing counts. One-minute thrashes after a brief acclimation in M9 buffer showed that thrashing of both *lam-3* RGE (114 thrashes per min, n=50) and *lam-3* ΔRGD (109 thrashes per min., n=50) are significantly higher than that of N2 (95 thrashes per min. (n=50), *p* < 0.0001) (Figure 1E). The number of thrashes between *lam-3* RGE and *lam-3* ΔRGD was not statistically different (*p* = 0.2145). Then, we also measured the overbending. The result showed increased hyper-contraction in *lam-3* RGD mutants, *lam-3* RGE (20 overbends per min., n = 50) and *lam-3* ΔRGD (22 overbends per min., n = 50), compared to N2 (16 overbends per min., n = 50, p < 0.05) (Figure 1F).

Bessou *et al.* found that mutants with muscle defects may show hypersensitive or resistant phenotypes to levamisole. This nicotinic drug targets levamisole-sensitive acetylcholine receptors (AChR) ion channels (Bessou *et al.* 1998). Levamisole stimulates the opening of the ion channels, promoting the entry of calcium ions, thus stimulating muscle contractions at neuromuscular junctions (Sloan *et al.* 2015). Levamisole response of the *lam-3* mutants (Figure 1G) revealed that, after 30-minute exposure to 100 µM levamisole, 76% of *lam-3* ΔRGD worms were paralyzed (n = 100), while the 35% of *lam-3* RGE (n = 105) were paralyzed. This result suggested that *lam-3* ΔRGD is hypersensitive to levamisole, while the *lam-3* RGE mutant was resistant to the chemical.

To identify additional defects of *lam-3* mutants, we also measured the touch sensitivity of the *lam-3* mutants. *C. elegans* has six touch receptor neurons anchored closely to the cuticle and surrounded by epidermal cells. These touch neurons allow worms to detect and produce responses to external forces (Chalfie and Sulston 1981). Stroking at either the anterior or posterior part of a responsive worm with the tip of human hair elicits a type of avoidance behavior (Krieg *et al.* 2015). Our results showed that both *lam-3* RGE (5%, n = 200, p > 0.011) and ΔRGD (5%, n = 200, *p* < 0.011) showed significant non-responsiveness to gentle touches compared to N2 (1.5%, n = 200), suggesting that the anchoring of touch neurons depends on the proper laminin function that ensures cell-matrix interaction.

We conclude that the mutations of *lam-3* RGD result in increased body thrashing, hyper-contraction, and defective mechanosensation. Responses to levamisole in *lam-3* RGD mutants confirm that *lam-3* is vital for muscle contraction of the body. In *C. elegans*, hyper-contraction and levamisole resistance are typical phenotypes of dystrophic muscles (Chaya *et al.* 2021; Ellwood *et al.* 2021). Mutations in *the dys-1*/dystrophin gene display hyperactive muscle contraction and levamisole resistance due to defective cholinergic transmission across neuromuscular junctions (NMJ) (Bessou *et al.* 1998). The *lam-3* RGD mutants may carry similar defects that lead to dystrophic muscle due to the defective NMJ. Future studies should further investigate the role of *C. elegans* ECM on the regulation of muscle functions.

## Methods

To delete the RGD motif in *lam-3* locus in chromosome I, we have identified an effective CRISPR site in the area of three amino acid numbers, 1462, 1463, and 1464, from the CRISPR guide RNA Selection Tool, http://genome.sfu.ca/crispr/search.html. According to the intended mutation, 94-mer repair DNA templates were designed and custom-made from IDT Inc., Coralville, IA, including all other PCR oligos in this study (Reagents section). Then, the mixture of template DNA (custom), crRNA (custom), tracr RNA (catalog no. 1072532), and Alt-R Cas9 (catalog no. 1081058) proteins are annealed at 37°C and micro-injected into the syncytial gonad arms of N2 animals (P0) with *dpy-10* crRNA as a co-CRISPR marker (Paix *et al.* 2015; Dickinson and Goldstein 2016). The F1 offspring of P0 worms is selected by Dpy phenotype and is subjected to PCR genotyping to identify worms carrying the desired mutations. Once the F1 mutants are isolated, F2 progeny are screened with wild-type specific or mutant specific primer sets to identify the homozygote alleles. Isolated PCR products were also sequenced to confirm the mutations. A thrashing assay was performed with young adult worms.

The one-minute counting of thrashing is conducted after one minute of acclimation in 40 μl of M9 buffer. Videos for thrashing assays were taken using mobile devices. The counting of thrashing and overbending was performed manually. Continuous and simultaneous bending of both the head and the tail towards either side of the axis was counted as one thrash. Touch sensitivity assays (gentle touch) were performed by stroking at the posterior to the pharynx and near the tip of the tails (Goodman and Schwarz 2003). The elicited response characterized as ‘responsiveness’ is a changing of movement from forward to backward. Non-responsiveness is the lack of characteristic responsiveness after stroking at both ends. A worm pick with fine hair attached to the end was used for the stroke. To characterize levamisole phenotypes, we performed a levamisole sensitivity assay by placing worms in 100 µM levamisole in M9 solution for thirty minutes and counting the number of paralyzed worms in ten-minute intervals. The paralysis was tested by a gentle stirring, the same tool aforementioned in the touch assay. The lack of muscle contraction in response to a mechanical stimulus is regarded as paralysis. All statistical analyses in this study were performed with Wilcoxon Analysis (Preisser *et al.* 2011) using JMP Pro 15.2.0 (SAS lab, Cary, PA).

## Reagents

**DNA template sequence for homology directed repair**

**Table:** 

Temp-LAM3RGD1464D	CAAGACACCCAAGGAATATATACCGTGGAACCTATACTTACCCGGCTGCAA TTAACATCCAAGAGGTTTCCCTTGACGTAGCTGTTCCTGAATC
Temp-LAM3RGE1464	CAAGACACCCAAGGAATATATATCCGTGGAACCTATACTTACCCGGCTCGA GGCGAAGCTATCAATATTCAAGAGGTTTCCCTTGACGTAGC

**crRNA for *lam-3* locus**

**Table:** 

LAM3RGD	ACCTACACATATCCAGCAAG	AGG (PAM sequence)

**co-CRISPR *dpy-10* crRNA**

ZQDP10A: GCTACCATAGGCACCACGAG

**Genotyping and Sequencing Primers**

**Table:** 

LAM3GRESEQF	GTCACTCTCCAGAGCTCACAC
LAM3RGESEQR	CAGTGCTCACAGAAATCACCG
LAM3RGDWTF	CTACACATATCCAGCAAGAGGTGAT
LAM3RGEF	CCGGCTCGAGGCGAA
LAM3RGD1464DF	TACTTACCCGGCTGCAATTAACATC

**Table:** 

**Strain**	**Genotype**	**Available from**
N2	Wild Type *Caenorhabditis elegans* Bristol Strain	CGC
BU1461	*lam-3 (kq1461)*	This study
BU1464	*lam-3 (kq1464)*	This study
